# The Effect of Acute Consumption of Energy Drinks on Blood Pressure, Heart Rate and Blood Glucose in the Group of Young Adults

**DOI:** 10.3390/ijerph15030544

**Published:** 2018-03-19

**Authors:** Dariusz Nowak, Michał Gośliński, Kamila Nowatkowska

**Affiliations:** Department of Nutrition and Dietetics, Faculty of Health Sciences, Ludwik Rydygier Collegium Medicum in Bydgoszcz, Nicolaus Copernicus University in Toruń, Dębowa 3, 85-626 Bydgoszcz, Poland; m.goslinski@cm.umk.pl (M.G.); kamilanowatkowska@interia.eu (K.N.)

**Keywords:** energy drinks, blood pressure, heart rate, blood glucose

## Abstract

Background: Energy drinks (EDs) are very popular among young people, who consume them for various reasons. A standard ED typically contains 80 mg of caffeine, as well as glucose, taurine, vitamins and other ingredients. Excessive consumption of EDs and accumulation of the above ingredients, as well as their mutual interactions, can be hazardous to the health of young adults. The purpose of this study was to assess the effect of acute consumption of energy drinks on blood pressure, heart rate and blood glucose. Methods: The study involved 68 volunteers, healthy young adults (mean age 25 years), who were divided into two groups: the first consumed three EDs at one-hour intervals, and the second drank the same amount of water. All participants had their blood pressure (BP)—systolic and diastolic (SBP and DBP)—as well as heart rate (HR) and blood glucose (BG) measured. In addition, participants could report any health problems before and after consuming each portion of ED. Results: In the above experiment, having consumed three portions of ED (240 mg of caffeine), the participants presented a significant increase in DBP (*p* = 0.003), by over 8%, which coincided with a lack of any significant impact on SBP (*p* = 0.809). No significant changes were noted in HR (*p* = 0.750). Consumption of EDs caused a significant increase (*p* < 0.001) in BG, by ca. 21%, on average. Some participants reported various discomforts, which escalated after 2 and 3 EDs. Conclusions: Acute consumption of EDs contributed to increased diastolic blood pressure, blood glucose and level of discomfort in healthy young people. Our results reinforce the need for further studies on a larger population to provide sufficient evidence.

## 1. Introduction

Energy drinks (EDs), the consumption of which is on the increase globally, are highly popular, particularly among adolescents [1,2] and college students [3,4,5]. There are various reasons why young people choose EDs. Some consume EDs to combat somnolence (67%), to gain more energy (65%) or while drinking alcohol at parties [3]. Some admit to drinking these beverages without any particular reason (21%) or when feeling exhausted (18%) [2].

Each ED may contain up to twenty ingredients, including: caffeine, taurine, glucuronolactone, glucose, sweeteners, B group vitamins and many others. A can of ED typically contains 80 mg of caffeine (up to 141 mg), which corresponds to or sometimes exceeds the caffeine content in a cup of coffee [6,7]. Some EDs contain peanuts, guarana, yerba mate, etc., which additionally raise the caffeine content, up to 300 mg [8,9]. Caffeine, if consumed in excess, may induce insomnia, increase arterial blood pressure (BP), and can later contribute to a higher risk of osteoporosis and cardiovascular diseases [10,11,12,13,14]. Moreover, the high glucose content in EDs is another health hazard. A can of ED, containing 8 floz (ca. 240 mL), will supply 21–34 g [15,16], or even 50–60 g, of glucose [17], which potentially promotes overweight and dental caries [16]. The world is now struggling with epidemics of obesity and type 2 diabetes. One of the reasons for this is the substantial increase in the consumption of beverages that contain sucrose and caffeine. Fructose, which—together with glucose—is a product of sucrose breakdown, has been associated with such health problems as body weight gain and insulin resistance [18]. People who consume EDs complain of feeling unwell, restless or anxious, as well as of suffering from insomnia, tachycardia and increased pulse rate [19]. Consumption of EDs can raise the risk of developing arterial hypertension and type 2 diabetes, while a high intake of caffeine lowers the sensitivity to insulin [1,14,20]. Recent studies have shown that consumption of ED, sugar-free ED or water + caffeine increases blood pressure compared to consumption of water [21]. Higher doses of caffeine consumed with a few EDs may cause an increase in BP and other parameters, e.g., blood glucose. Therefore, further studies in this field are needed.

The purpose of this study was to assess the effect of acute consumption of energy drinks (EDs) on arterial blood pressure (systolic and diastolic: SBP and DBP), heart rate (HR) and blood glucose (BG) in the context of potential risk of cardiovascular diseases.

## 2. Materials and Methods

### 2.1. Subjects

The study was carried out on a group of volunteers, students of a private college (Bydgoszcz, Poland). Healthy volunteers (aged over 18 years) were recruited for the study. Exclusion criteria were: cardiovascular disease, any medication affecting the cardiovascular system, diabetes, other chronic diseases, pregnancy, lactation, and regular alcohol intake. All subjects signed an informed consent after reading the purpose and schedule of the research. Originally, 72 volunteers were recruited, and they were divided into two groups: the first consuming energy drinks (ED group), and the second drinking water (Control group). The assignment of participants to each group was random, aided by a random number generation formula (an MS Excel application). Before joining the study, the participants received verbal instructions. First, the participants were advised not to take alcohol for at least 24 h prior to the study date. Additionally, they were asked not to consume any caffeine-containing products, such as tea, chocolate or cola drinks, for at least 12 h before the study. Moreover, subjects were requested not to do any excessive physical activity prior the test. Four subjects from the Control group were withdrawn just before the study for various reasons (did not meet the recommendations or were afraid of blood tests). Finally, 68 participants (mean age 25 years) joined the study: 36 in the ED group and 32 in the Control group.

### 2.2. Study Protocol

The study was carried out in the winter season (between December 2016 and March 2017). All study participants provided informed consent for the test and their baseline characteristics were collected. Before the experiment, the participating students received a survey questionnaire, which inquired about consumption of different sources of caffeine and sugar over the previous 12 h. Next, each participant from the experimental group consumed three EDs, each at 250 mL (750 mL in total), at one-hour intervals. The study used one of the most popular energy drinks purchased at the local market. Each ED contained 80 mg of caffeine and over 20 g of sugar, as well as other ingredients, as shown in Table 1.

Having consumed each ED, the participants could report any discomfort they felt. The Control group consumed 250 mL of spring water (750 mL of water in total), also at one-hour intervals. The use of water as a control was similar to the studies by Grasser et al. and Worthley et al. [13,22]. The EDs and water used for the study were at room temperature. Each participant had their blood pressure (SBP and DBP) and heart rate (HR) measured seven times, i.e., before having the first ED and after 0.5 h and 1 h following the consumption of each 250 mL portion. BP was measured with a Hartmann Tensoval Comfort sphygmomanometer (previously validated) after the participants had been seated and had rested for at least 5 min. BP and HR measurements were done in accordance with the guidelines of the European Society of Hypertension and European Society of Cardiology [23], and the recommendations by Kallioinen et al. [24]. All tests were performed twice at 1–2 min intervals at room temperature on the arm of the non-dominant hand. In the case of large differences, the measurement was repeated once more. The blood glucose level was measured (with a glucometer Optium XIDO, Abbott Diabetes Care Ltd., Witney, UK) before having the first ED, and 1 h after consuming the third ED. Analogous measurements were taken on participants assigned to the Control group, although BP and HR were measured 1 h after consumption of each portion (250 mL) of water. The study was performed in accordance with the Helsinki Declaration, and the protocol was approved by the Bioethical Committee.

### 2.3. Statistical Analysis

All the results were statistically analyzed by calculating the mean and standard deviation. The interpretation of the results was performed in MS Excel 2010 Analysis ToolPak software (Microsoft, Redmond, WA, USA), with one-way analysis of variance (ANOVA) and the Tukey’s test as the post hoc test. *p*-Values lower than 0.05 were considered as significant.

## 3. Results

A total number of 68 volunteers was recruited for this study, including 53 (78%) female students. Baseline characteristics of the study participants are shown in Table 2. The majority of the participants had a normal BMI. For example, 83% in the group consuming EDs had a normal BMI (18.5–24.9). 5 subjects (14%) were overweight, and 1 subject was underweight. The percentage of participants with a normal BMI in the group consuming water was similar, and equaled 84%.

**Figure 1:**
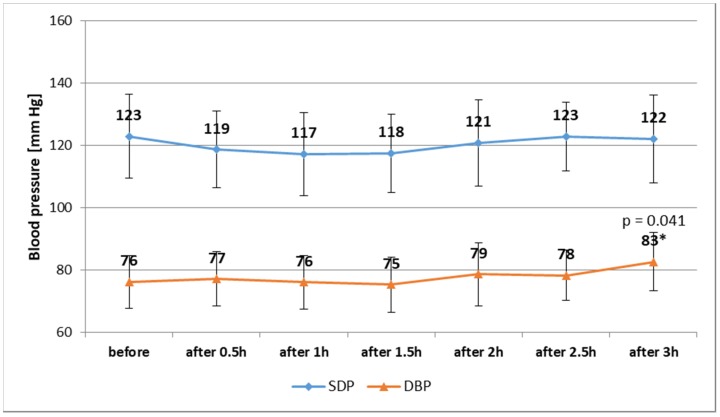
Changes in SBP and DBP of participants—ED group. Data are mean ± SD (*n* = 36); * Statistical significance (at *p* < 0.05) was only stated between 2.5 and 3 h; SBP—systolic blood pressure; DBP—diastolic blood pressure.

For the ED participants, our analysis of changes in arterial BP did not show statistically significant differences (*p* = 0.809) regarding SBP (Figure 1; Table 3). However, having consumed 3 EDs, 14 subjects (39%) had increased SBP (by min. 0.7%, max. 30.8%) compared to SBP measured before consumption. The consumption of 3 EDs caused a statistically significant (*p* = 0.003) increase in DBP by over 8% (Figure 1; Table 3). An increase in DBP was observed in as many as 23 (64%) subjects (min. 1.1%, max. 48.5%).

**Figure 2:**
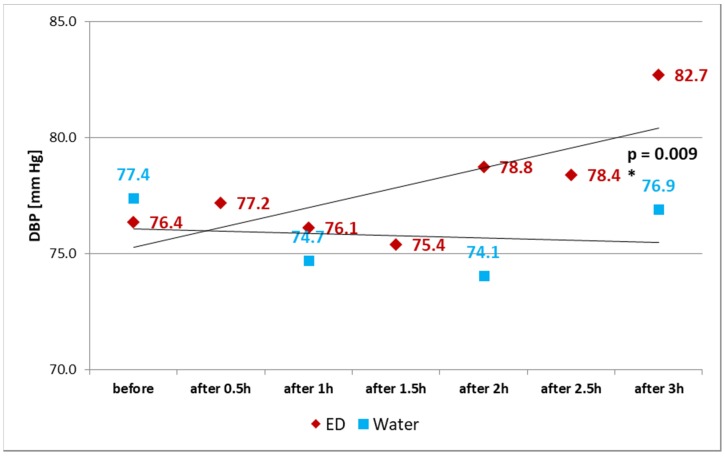
Changes in DBP after consuming EDs and water. * Statistical significance (at *p* < 0.05) between ED and water. DBP—diastolic blood pressure.

The second group, consuming water (Control group) instead of EDs, did not demonstrate a statistically significant increase in SBP (*p* = 0.863) or DBP (*p* = 0.820) (Table 3). Statistical analysis showed significant differences (*p* = 0.009) in trends of changes in DBP between participants consuming EDs or water, which are presented in Figure 2. There were no significant differences (*p* = 0.874) in changes of SBP.

Consumption of ED did not have a statistically significant (*p* = 0.750) effect on increasing the HR (Table 2). No significant changes (*p* = 0.491) within these parameters were observed among the control group participants, who consumed water.

**Figure 3:**
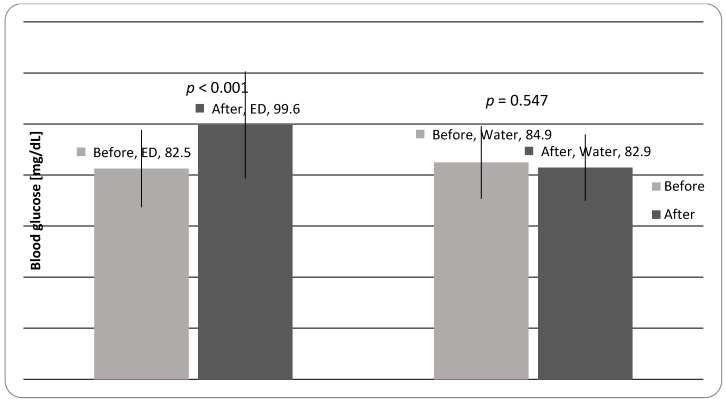
Values of blood sugar (mean) in the study participants before and after 3 EDs/water consumption.

Consumption of EDs had a significant (*p* < 0.001) effect on the increase of BG among the research participants (Table 3, Figure 3). An average increase in the glucose content in blood was 20.7%, although there were 11 subjects out of the total number of 36 group members (31% of the whole group) whose BG increased by over 40%, and in 2 subjects, the level of BG doubled. After 3 EDs, 19 subjects were diagnosed as having a BG above 100 mg/dL (min. 103; max. 163). No significant changes (*p* = 0.547) were observed among the control subjects with respect to their BG.

The research participants who consumed EDs were asked about their well-being after they had drunk each portion of ED. The results are collated in Table 4. Participants consuming EDs most often reported the feeling of excitation, headache, somnolence, malaise and others (mostly irritability and xerostomia). Following the first portion of ED, most complaints reported were about excitation; after the second portion—somnolence, headache, malaise and others; while after the third portion—excitation, malaise and others.

## 4. Discussion

Energy drinks are very popular among young adults, which exposes them to higher risk of negative effects of EDs ingredients. Numerous investigations have revealed a link between EDs and their ingredients and acute arterial hypertension. This effect is associated with hemodynamic changes induced by consumption of caffeine. High caffeine intake may increase renin, catecholamine and dopamine levels in blood plasma. These substances stimulate the central nervous system, which raises the BP and HR [14]. EDs are also sources of high levels of sugar. Therefore, the purpose of this study was to assess the effect of acute consumption of EDs on arterial blood pressure (SBP, DBP), HR and BG, i.e., some of the parameters which may indicate a potential risk of cardiovascular diseases. The influence of EDs ingestion was considered in the context of cardiovascular (BP, HR) and metabolic (BG) changes.

### 4.1. Cardiovascular Changes

In our study, following the consumption of three EDs (240 mg of caffeine) the participants were found to present a significant (*p* = 0.003) increase in DBP, by over 8%, while no significant (*p* = 0.809) effect on SBP was detected. The literature shows various data related to the impact of EDs on BP, although they mostly concern the acute impact of one ED. Franks et al. demonstrated that consumption of energy drink had a stronger effect on an increased BP than an aqueous solution with an identical content of caffeine. The researchers suggested that the increase in BP they observed may have been stimulated by the presence of such ingredients in EDs as inositol or taurine [25]. On the other hand, Miles-Chan et al. showed the impact of caffeine on increase in BP. Students who consumed 355 mL EDs (with sugar or sugar-free) as well as water with caffeine (120 mg) had 3–4 mm Hg higher BP in comparison to consumption of pure water [21]. Worthley et al. found a significant (*p* < 0.05) increase in BP (about 4%) after the consumption of 250 mL glucose-free ED (80 mg of caffeine) compared with 250 mL carbonated water (control) [22]. Likewise, in a study conducted on 25 healthy university students, all with a normal BMI, it was demonstrated that 2 h after the consumption of 355 mL of ED (114 mg of caffeine) increased SBP and DBP by 5.2 and 6.1 mm Hg, respectively. There was no effect in the control group consuming 355 mL of tap water [13]. In our experiment, the increase in DBP was similar (by 6.4 mm Hg), but it occurred after 3 EDs had been consumed. Another study of 50 young, healthy subjects (mean age 25 years, BMI 25.6 kg/m^2^, 60% males) showed that 1 and 2 h after consumption of 355 mL of a very popular ED, SBP increased significant (*p* < 0.05) from 112 to 123 and 121 mm Hg, respectively, and DBP rose from 73 to 77 and 76 mm Hg, respectively [26]. A review paper by Higgins et al. shows that a typical increase in SBP and DBP of healthy participants 1–2 h following consumption of ED is approximately 6–10 mm Hg and 3–6 mm Hg, respectively [14]. In yet another experiment, 24 h after consumption of 250 mL ED (80 mg of caffeine), the average SBP and DBP were significantly higher in the ED group than in the control (132.2 vs. 117.4 mm Hg, 73.6 vs. 68.2 mm Hg, respectively) [25]. In contrast, Ragsdale et al., in a placebo-controlled study, reported no change in BP throughout a 2h test period after taking 250 mL of ED [27]. Furthermore, Hajsadeghi et al. concluded that a 250 mL dose of ED (80 mg of caffeine) had no statistically significant effect on BP (SBP and DBP) measured in 44 healthy adults 0.5, 2 and 4 h after consumption [28]. Furthermore, Steinke et al. evaluated a long-term consumption of EDs. In their study, comprising 15 healthy young adults, who were given 2 cans of ED (500 mL) daily for a week, a significant increase in SBP was noted on day 1 and 7, by 7.9% (*p* = 0,006) and by 9.6% (*p* < 0.001), respectively, while DBP rose by 7.0% on day 1 and by 7.8% on day 7 [29].

In our research, no significant (*p* = 0.750) changes in heart rate (HR) were observed following the consumption of EDs. Similar findings were reported by Worthley et al. [22]. Contrary results were obtained by Steinke et al., who demonstrated an increase in HR by 7.8% within 4 h of ED consumption (500 mL) [29]. Another study, which involved 12 healthy and physically active participants, showed that after drinking an ED (containing 1 or 3 mg of caffeine per kg of body weight), the HR of the participants increased by 2 or 5 beats/min relative to the placebo group (a caffeine free beverage) [30]. On the other hand, Hajsadeghi et al. showed statistically significant HR decline (*p* = 0.004) 4 h after consumption of 250 mL of ED [28]. In summary, our studies did not show a significant increase in HR, although Higgins et al. noted that acute consumption of EDs has been associated with small but significant increases in heart rate [14].

### 4.2. Metabolic Changes

Our results have demonstrated that consumption of EDs significantly (*p* < 0.001) increased the level of blood glucose (BG), by an average of 21%. One ED supplies over 20 g of glucose, which can have adverse health effects. It may be speculated that glucose in ED could affect the cardiovascular system, as the intake of calories is generally accompanied by an increase in HR, cardiac output, and pulmonary ventilation rate [31]. Moreover, EDs appear to provide the consumer with a blood glucose increase that may stimulate an insulin response and enhance the glucose pool in body cells [27]. Consumption of high levels of sugar causes various detrimental effects on health, especially inducing insulin resistance, which is closely associated with the development of metabolic disorders such as obesity or type 2 diabetes [32,33]. In addition, high levels of blood glucose may cause oxidative stress through the overproduction of reactive oxygen species [34]. Furthermore, some studies have proved that caffeine may play an important role in the regulation of insulin release and related metabolic disorders. González-Domínguez et al. showed that healthy young adults who consumed sugar-sweetened drinks with caffeine had a significant increase in blood glucose and insulin levels after 20–30 min. The authors concluded that adverse effects may be the result of synergic effect of caffeine and sugar [33].

### 4.3. Discomfort after EDs Consumption

Consumption of EDs can also result in feeling worse. Participants of our study most often reported symptoms including: excitation, headache, malaise and others, e.g., irritability or xerostomia. It is worth noting that after ingestion of first ED, the most frequently reported discomfort was excitation. Next, after the second portion of ED, the number of excitation symptoms decreased, while somnolence, headache and others (irritability, xerostomia, stupor) increased. This could probably be due to the effect of caffeine intake. The third dose of ED resulted in an increase of cases of excitation, stomachache and shaking hands. Among malaise and other discomforts, participants most often reported xerostomia, which could be a result of the dehydrating effect of caffeine. Furthermore, cardiovascular symptoms, such as light chest pain, palpitation or weakness, were noted. Other authors also indicated various adverse symptoms after consumption of EDs. Salinero et al., in their study concerning consumption of caffeinated ED (3 mg caffeine/kg body mass) by athletes (*n* = 90), reported side effects such as nervousness and insomnia in comparison to consumption of a placebo (decaffeinated ED, 0 mg/kg) [12]. In another study, all participants reported negative symptoms (palpitation, anxiety, insomnia) three hours after drinking caffeine-rich ED [35]. Costa et al. stated that excessive caffeine (from EDs) intake induces palpitations, and increases blood pressure, as well as other discomforts (anxiety, insomnia, vomiting, irritability and nervousness) [36]. In our previous investigation (questionnaire study, *n* = 488), about 10% of young adults complained of such health problems occurring after consumption of EDs, including: abdominal pains, arrhythmia and nausea [37]. A survey done by Rahamathulla showed that among students declaring consumption of EDs (*n* = 274), the most common side effects were headache (32%), stomachache (21%), increased urination (11.2%), restlessness (9.7%), hypertension (9.1%), tingling (7.1%) and nervousness (6%) [38].

Summarizing, regular intake of EDs may raise the risk of cardiovascular diseases because of their content of caffeine, sugar and other ingredients, which can increase arterial BP, HR and BG levels. Acute ingestion of caffeine or caffeinated EDs has been shown to increase blood pressure, which may lead to an acute adverse hemodynamic profile with an augmented cardiac workload and diminished cerebral blood flow velocity. These adverse changes are most likely caused by caffeine or by the effect of an interaction between caffeine and glucose on the cardiovascular system [31]. Literature data reported that consumption of ED caused the same increase in BP as caffeine, although this occurs through different hemodynamic pathways. EDs mainly impact cardiac parameters, while caffeine induces vascular effects, in particular [21]. Our study showed significant increase in DBP and BG, which was probably the effect of synergism of the caffeine and sugar content in our ED. We did not observe statistically significant changes in BP and HR in the group consuming water. On the other hand, Monnard and Grasser reported significant decrease in BP and HR 0.5 hour after consumption of 355 mL tap water [39]. Furthermore, it should be underlined that the high content of sugar in EDs, combined with their popularity among young people, could promote overweight, obesity, dental caries and other health problems. Various discomforts after consumption of EDs were noted in our study. Their number increased following each ED, which demonstrates the cumulative effect of ED ingredients. Another problem is mixing EDs with alcohol, which has been demonstrated in our previous studies [2].

## 5. Conclusions

We have demonstrated that acute consumption of energy drinks (EDs) contributes to an increase in diastolic blood pressure (DBP) and blood glucose (BG) in young, healthy participants. We did not detect any significant effect on systolic blood pressure (SBP) and heart rate (HR) after consumption of 3 EDs. No statistically significant changes in BP and HR were observed in the Control group consuming water. Apart from cardiovascular and metabolic changes, a lot of different types of discomfort were reported by participants, which increased following each ED. The most frequently reported symptoms were excitation, headache, somnolence, malaise and others. In conclusion, acute ingestion of EDs may lead to various health problems associated with the synergic effects of over-consumption of caffeine and sugar. Further studies on a larger population are needed to provide sufficient evidence.

## Figures and Tables

**Table 1:** Composition details of consumed energy drink.

Ingredients	Unit	Per 100 mL
Water		
Energy	kcal	46
Protein	g	0
Total lipid	g	0
Carbohydrates	g	11
In it sugar	g	11
Minerals:		
sodium, Na	mg	70
Vitamins:		
niacin	mg	7.0
pantothenic acid	mg	2.0
vitamin B-6	mg	0.7
vitamin B-12	µg	0.5
Others:		
Caffeine	mg	32
Taurine	mg	400
Inositol	mg	20
Acidity regulator		
sodium citrate		
citric acid		
Carbon dioxide		
Flavor		
Color		
riboflavin (E101)		
caramel (E150d)		

**Table 2:** Baseline characteristics of the study subjects.

Parameter	ED Group (*n* = 36)	Control Group (*n* = 32)
Sex		
Female	28	25
Male	8	7
Age, years	24.8 ± 6.9 ^a^	25.0 ± 7.3 ^a^
Height (m)	1.70 ± 0.08 ^a^	1.71 ± 0.08 ^a^
Weight (kg)	65.4 ± 11.9 ^a^	65.1 ± 11.9 ^a^
BMI		
>24.9	5	4
18.5–24.9	30	27
<18.5	1	1

Values are the mean ± standard deviation; ^a^ Different letters in the same row indicate statistical significance (at least *p* < 0.05).

**Table 3:** Blood pressure (SBP, DBP), heart rate (HR) and blood glucose (BG) of participants.

Parameter	ED Group (*n* = 36)			Control Group (*n* = 32)		
	Before EDs	After 3 EDs	*p*	Before Water	After 3 Waters	*p*
SBP (mm Hg)	123.0 ± 13.4	122.2 ± 14.0	0.809	121.2 ± 12.3	121.7 ± 10.7	0.863
DBP (mm Hg)	76.4 ± 8.5	82.7 ± 9.3	0.003	77.4 ± 7.8	76.9 ± 8.6	0.820
HR (beats per minute)	79.7 ± 13.6	78.7 ± 12.1	0.750	74.0 ± 9.6	75.8 ± 10.9	0.491
BG (mg/dL)	82.5 ± 15.1	99.6 ± 21.0	<0.001	84.9 ± 14.2	82.9 ± 13.0	0.547

Data are mean ± SD; Statistical significance at *p* < 0.05. SBP—systolic blood pressure; DBP—diastolic blood pressure; HR—heart rate; BG—blood glucose.

**Table 4:** Symptoms reported by the research participants during the consumption of EDs *.

Symptoms (Discomforts)	after 1 ED	after 2 ED	after 3 ED	*n*
headache	3 (8%)	7 (19%)	3 (8%)	13
light chest pain	-	-	2 (5%)	2
stomachache	-	2 (5%)	5 (14%)	7
excitation	9 (25%)	6 (17%)	8 (22%)	23
anxiety	2 (5%)	-	-	2
shaking hands	-	1 (3%)	4 (11%)	5
somnolence	-	8 (22%)	5 (14%)	13
malaise and others	3 (8%)	9 (25%)	10 (28%)	22

* Each participant could report more than 1 discomfort after consuming ED.
